# Transcriptional reprogramming in oral squamous cell carcinoma

**DOI:** 10.1038/s41598-025-01364-w

**Published:** 2025-05-25

**Authors:** Xianyang Cheng, Shan Shen

**Affiliations:** 1 School of Stomatology, Jinan University, Guangzhou, 510000 China; 2 Department of Stomatology, The First Affiliated Hospital of Jinan University, Guangzhou, 510000 China

**Keywords:** Oral squamous cell carcinoma, RRA, WGCNA, Key gene, Biological function, Biotechnology, Cell biology, Computational biology and bioinformatics, Medical research

## Abstract

Oral squamous cell carcinoma (OSCC) is a prevalent form of cancer globally. This disease is characterized by its complex genetic underpinnings, involving the intricate regulation of multiple genes. Genetic factors influence cellular processes such as growth, differentiation, and apoptosis of oral mucosal cells, thereby promoting or inhibiting tumor formation and progression. Furthermore, environmental factors—including smoking, alcohol consumption, and human papillomavirus (HPV) infection—can significantly increase the risk of developing OSCC. These external influences can impact the disease in several ways. Delayed clinical detection and the absence of specific biomarkers, coupled with expensive treatment alternatives, contribute to poor prognoses among OSCC patients. Thus, identifying OSCC biomarkers has become imperative. This study investigates publicly accessible sequencing data of oral mucosal tissues from four distinct datasets—GSE23558, GSE30784, GSE36090, and GSE51010—archived in the Gene Expression Omnibus (GEO) database. By analyzing these datasets, which encompass a range of genetic profiles and experimental conditions, the study seeks to uncover critical biomarkers and molecular pathways involved in the early stages of OSCC development. The primary objective is to identify pivotal genes linked to the onset of OSCC. The findings provide preliminary evidence for therapeutic targets in OSCC and may serve as a robust foundation for subsequent biological research endeavors.

## Introduction

OSCC is a type of malignant tumor that arises from the squamous epithelial cells lining the oral mucosa. This carcinoma develops within the outermost layer of the oral mucosal tissue, which is primarily composed of squamous cells—flat, thin cells that form the surface layer of the mucous membranes. As OSCC progresses, it invades deeper into the surrounding tissues and structures, leading to aggressive growth and potential metastasis. OSCC ranks as one of the most frequently encountered malignant tumors within the oral cavity. This cancer often originates from several potential sites, such as the tongue, the floor of the mouth, the buccal mucosa (inner cheeks), and the lips. Its development has been closely linked to certain high-risk lifestyle behaviors. Specifically, the habitual use of tobacco, excessive alcohol consumption, and betel nut chewing have all been identified as significant contributors to the increased risk of OSCC^1,2^. OSCC occurs globally, but the prevalence varies significantly in different regions. For example, the prevalence is higher in Asia and South America, while it is relatively lower in North America and Europe^3,4^. The incidence of oral squamous cell carcinoma in men is approximately twice that in women, mainly due to the fact that smoking and drinking are more common among men, and these two factors are the primary risk factors for this disease^5–7^. OSCC occurs mainly in the middle-aged and older population, especially those over 50 years of age. However, in recent years, it has been found that the incidence of OSCC has also increased in younger people, in part related to an increase in HPV infection. Certain specific strains of the human papillomavirus (HPV), notably HPV-16 and HPV-18, play a significant role in the development of OSCC. These high-risk types of HPV are deemed crucial factors in the pathogenesis of this malignancy due to their ability to integrate into the host genome and express viral oncoproteins, particularly E6 and E7. The E6 protein promotes the degradation of the tumor suppressor protein p53, thereby inhibiting apoptosis and allowing the accumulation of genetic mutations. Simultaneously, the E7 protein inactivates the retinoblastoma (Rb) protein, leading to uncontrolled cell cycle progression and cellular proliferation. These particular types of HPV are deemed crucial factors in the pathogenesis of this malignancy^8,9^.

Early symptoms of squamous oral cancer are not obvious and may manifest as chronic ulcers, plaques or nodules of abnormal color. These symptoms are often mistaken for oral inflammation or ulcers, thus often leading to late diagnosis. Advanced oral squamous cell carcinoma (OSCC) poses significant treatment challenges, characterized by its resistance to therapy and high likelihood of recurrence. As the disease progresses, it becomes increasingly difficult to manage effectively, leading to a more complex and intensive treatment regimen. For early-stage oral squamous cell carcinoma (OSCC), surgical resection is a widely used treatment approach. This method involves the removal of the tumor and surrounding tissue to achieve a cure, as the cancer is localized and has not yet spread significantly. In contrast, the management of advanced OSCC often requires a more comprehensive strategy. In such cases, radiotherapy and chemotherapy are frequently employed in conjunction with surgical treatment to enhance patient outcomes. Radiotherapy uses high-energy radiation to target and destroy cancer cells, while chemotherapy involves the use of cytotoxic drugs to eliminate cancer cells throughout the body. Both treatments are aimed at reducing tumor size, targeting any remaining cancerous cells, and addressing potential metastases. This multi-modal approach not only helps in controlling the progression of the disease but also in improving overall prognosis and survival rates for patients with more advanced stages of OSCC^10,11^. In recent years, targeted therapy and immunotherapy have emerged as promising treatment options for advanced or refractory OSCC, offering new hope for patients who may not be suitable candidates for traditional chemotherapy or radiotherapy^12,13^. The prognosis for patients with OSCC is often poor, primarily due to the limited availability of early diagnostic tools and reliable biological markers in clinical settings^14,15^. In this study, we employed bioinformatics analysis to integrate and analyze sequencing data with the goal of identifying key genes associated with OSCC. By applying advanced computational methods to large-scale genomic datasets, we aimed to uncover critical genetic markers that are pivotal in the development and progression of OSCC. This approach seeks to enhance our understanding of the molecular underpinnings of the disease and identify potential biomarkers for early diagnosis. Additionally, the insights gained from this analysis are intended to inform the development of targeted therapeutic strategies, offering a solid foundation for both early detection and the creation of novel treatment options for OSCC.

## Materials and methods

### Data processing method and software sources

The original data for this study were obtained from the Gene Expression Omnibus (GEO) database^16^, and consist of transcriptome gene expression matrices. These datasets include four distinct gene expression profiles: GSE23558, GSE30784, GSE36090, and GSE51010. Each dataset represents a cohort of OSCC cases alongside healthy control samples.

Data processing and analysis were performed using R statistical software (version 4.4.0, available at https://www.r-project.org/). In order to deal with batch effect of multiple data sets, we used ComBat method in sva R package to remove batch effect of multiple data sets. The ComBat approach adjusts for systematic differences between batches by utilizing a Bayesian framework to ensure that data from different batches is comparable for downstream analysis. This method can effectively correct batch effects caused by technical or experimental factors in the data, thereby reducing the interference of these non-biological variations on the results^17^. Differential gene expression analysis was carried out with the “limma” package^18^, allowing us to identify genes with significant expression changes. For the enrichment analysis of differentially expressed genes (DEGs), we employed the “clusterProfiler” package^19^, which facilitated the exploration of biological pathways and functions associated with the DEGs. Visualization of the selected DEGs was achieved through the “pheatmap” package, which provided clear heatmaps of gene expression patterns. Additionally, gene co-expression analysis was conducted using the “WGCNA” package^20^, enabling us to identify clusters of genes with correlated expression profiles and explore their potential functional relationships.

### RRA analysis

Robust rank aggregation (RRA) is a widely utilized method in bioinformatics and computational biology, designed to synthesize rankings derived from various sources into a cohesive consensus list^21^. This approach is especially useful when working with disparate datasets or varying methodologies, which may each introduce their own biases or sources of noise. By systematically combining these rankings, RRA method improves the overall reliability of the final integrated ranking. RRA achieves this by effectively mitigating individual dataset inconsistencies and noise, thereby offering a more precise and reliable summary of shared insights across multiple datasets. This enhanced accuracy in the integrated ranking allows for a more robust interpretation of the data, reflecting a consensus that is less affected by the inherent variability and potential biases of the individual datasets.

In this study, we utilized the RRA package to integrate and analyze data from four separate datasets. To identify significant genes, we established criteria that included a p-value threshold of less than 0.05 and an absolute log2 fold change (FC) greater than 0.585. This stringent selection process enabled us to accurately identify key genes associated with OSCC. By applying these criteria, we were able to highlight genes with substantial and statistically significant expression changes, thereby deepening our understanding of their potential roles in OSCC. This robust approach not only clarified the relevance of these genes in the context of the disease but also laid the groundwork for future biological research aimed at exploring their functional implications and therapeutic potential.

### WGCNA analysis

In our gene co-expression analysis, we utilized the Weighted Gene Co-expression Network Analysis (WGCNA) package to construct gene regulatory networks that provide insights into underlying disease mechanisms. To ensure the quality and reliability of our analysis, we calculated Pearson correlation coefficients to detect and address any outliers present in the data, thereby safeguarding its integrity. By applying a soft threshold of 0.9, we carefully selected nodes from the gene expression profiles, optimizing the network structure to maintain a scale-free topology. Subsequently, we delved into the intricate relationships between specific gene expression profiles and characteristic genes, leveraging this network to cluster genes exhibiting similar expression patterns into distinct modules. This systematic approach allowed us to unveil potential regulatory networks and uncover meaningful associations between genes, shedding light on the intricate molecular mechanisms underlying the pathogenesis of the disease.

### GO and KEGG analysis

The Gene Ontology (GO) functional enrichment analysis encompasses three primary components: Molecular Function (MF), Biological Process (BP), and Cellular Component (CC). These components evaluate distinct facets of gene function and their localization within biological systems. To perform the GO term enrichment analysis, we used the “enrichGO” R package^19^, which identifies statistically significant GO terms linked to the provided gene set. This analysis highlights relevant biological processes, molecular functions, and cellular components associated with the genes under investigation. Additionally, for a more comprehensive understanding of functional roles, we employed the “enrichKEGG” R package^19^ to conduct functional enrichment analysis based on the Kyoto Encyclopedia of Genes and Genomes (KEGG) database. This approach provided insights into the pathways and biological networks that are pertinent to the genes in our study, enhancing our grasp of their functional significance and involvement in various metabolic and signaling pathways. This approach helps elucidate the biological pathways and processes in which the identified genes are involved, providing insights into their potential roles and interactions within cellular systems. Overall, these analyses enable a comprehensive understanding of gene functions, biological processes, cellular components, and pathway associations, crucial for interpreting the molecular mechanisms underlying complex biological phenomena.

### Ethical declaration

All data utilized in this research were sourced from publicly available databases. This study does not involve experiments or investigations conducted on animals or humans. The use of existing datasets ensures compliance with ethical standards and regulations governing research involving human and animal subjects, emphasizing the reliance on secondary data analysis for scientific inquiry.

## Results

### Data details

This study incorporates a total of four datasets, each described in detail in Table 1. The characteristics and specifics of these datasets are comprehensively outlined, providing essential information for understanding their relevance and contribution to the research conducted.

**Table 1:** Details of the included datasets.

GSE ID	Partipants	Species	Analysis type	Year
GSE23558	27cases and 5control	human	Array	2011
GSE36090	10cases and 3control	human	Array	2012
GSE30784	167cases and 45control	human	Array	2011
GSE51010	48cases and 8control	human	Array	2014

### Overall workflow

**Fig. 1:**
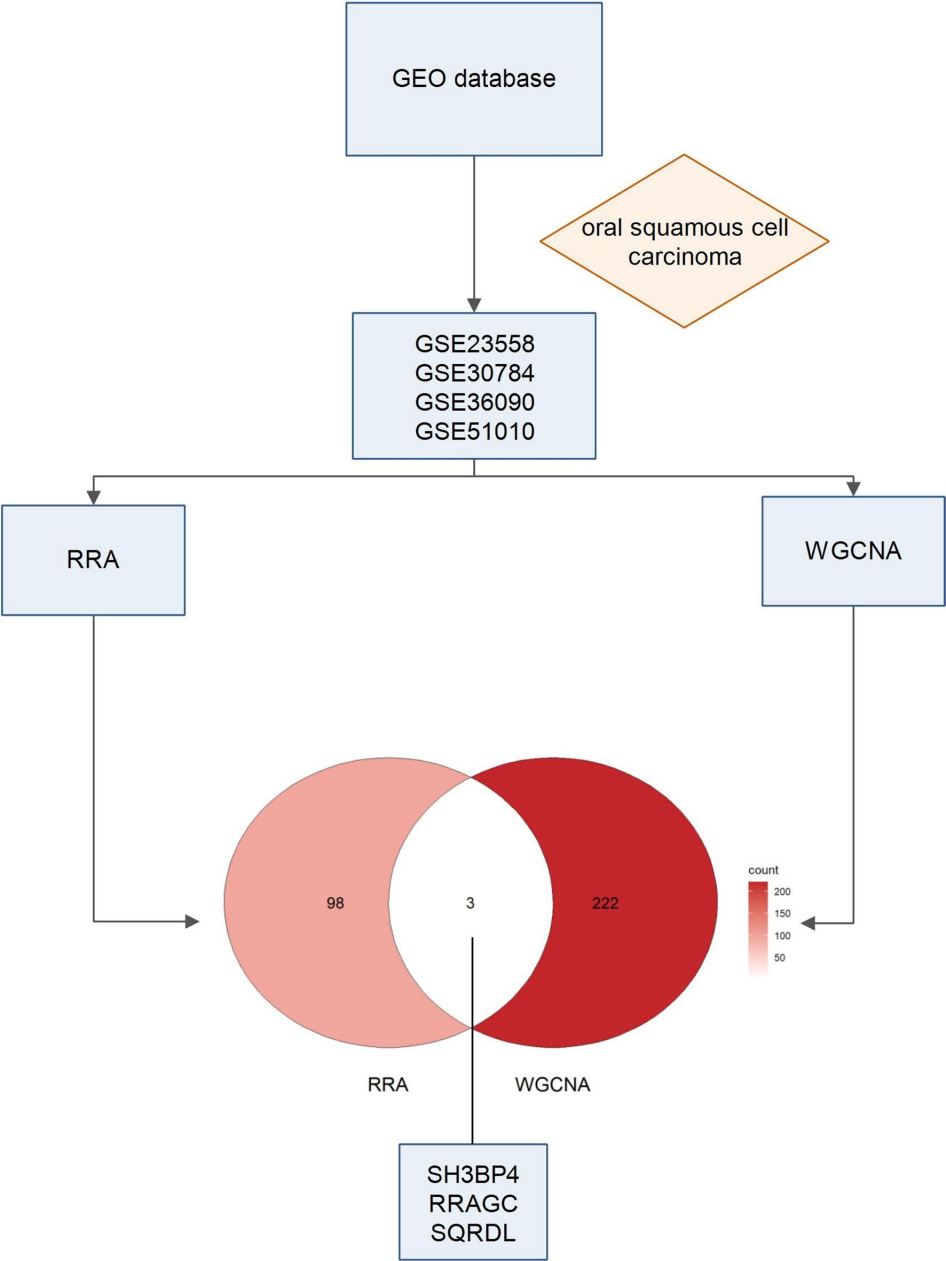
Workflow of this study. Above all, we searched the GEO database for datasets related to OSCC, and eventually identified four datasets. We conducted RRA and WGCNA on these two datasets. Finally, by intersecting the results of both analyses, we identified 3 hub genes.

In this study, we obtained raw gene expression matrices of two datasets from the GEO database. Each dataset underwent RRA and WGCNA analyses separately. WGCNA analysis identified 225 differentially expressed genes, which were intersected with 101 differentially expressed genes identified by RRA, resulting in a final set of 3 genes. These 3 genes, namely SH3BP4, RRAGC, and SQRDL, were identified as key genes. Details of the specific workflow are depicted in Fig. 1.

### Data pre-processing

**Fig. 2:**
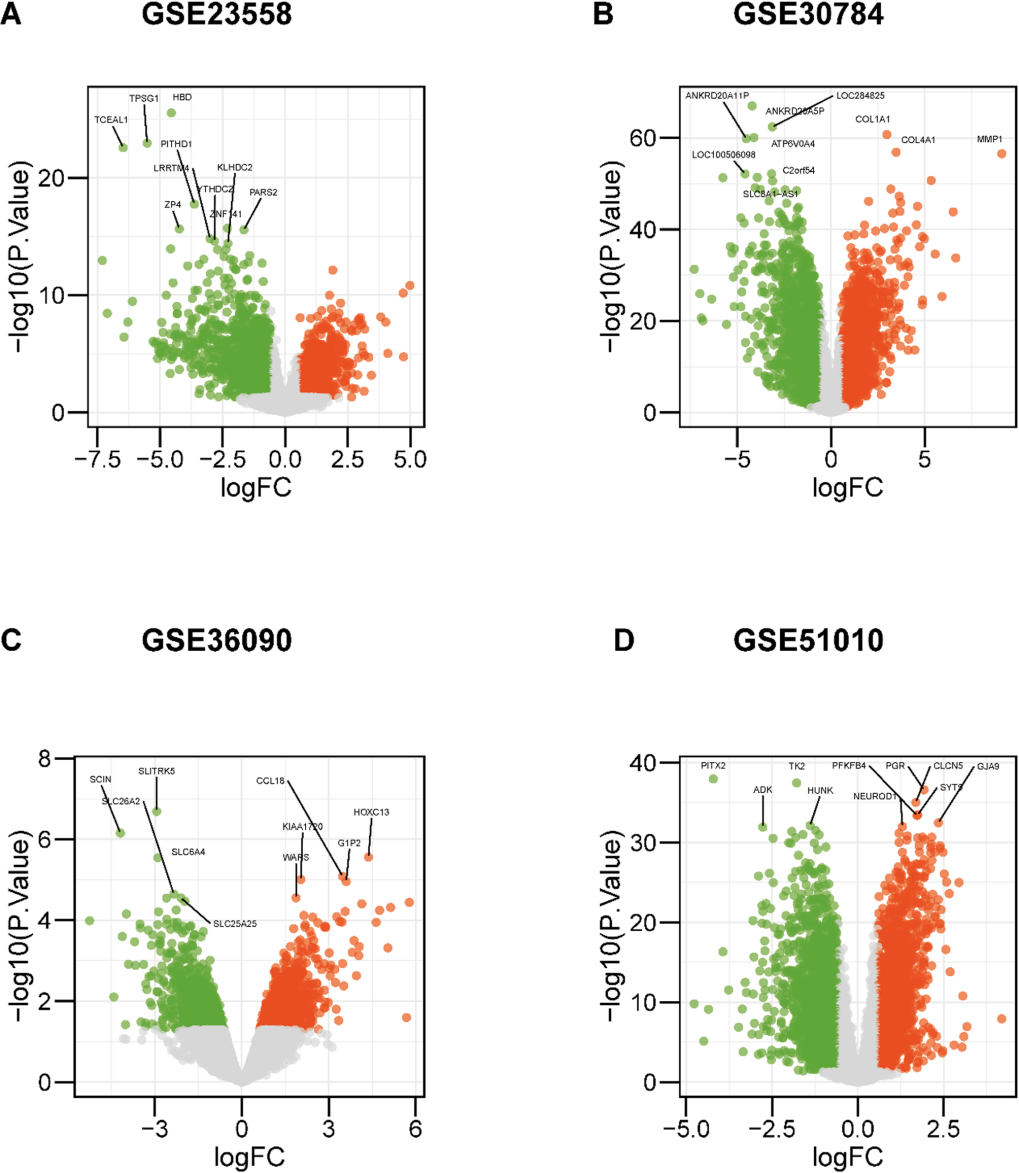
Difference analysis of each dataset. First, we used the limma package to normalize the individual datasets and then performed a difference-in-difference analysis to flag the top 10 genes with the most significant *P*-values.

**Fig. 3:**
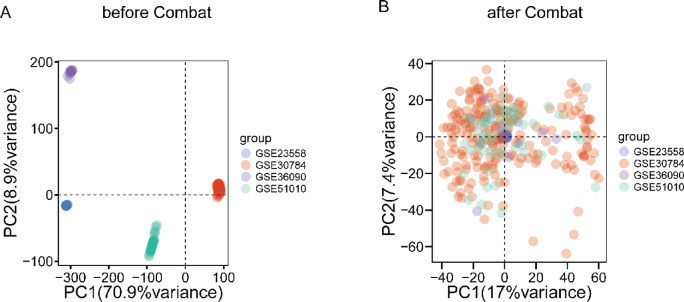
Normalization of datasets. (**A**) the initial dataset before addressing batch effects, and (**B**) the dataset post-batch effect removal. To mitigate batch effects, the Combat function from the “sva” R package was applied. By employing Combat, variations introduced by batch-specific factors were minimized, allowing for more accurate analyses and robust conclusions to be drawn from the normalized dataset.

The microarray datasets were first normalized using the quantile normalization method to ensure consistency across samples. Differential analysis was then performed individually on each of the four datasets, leading to the identification and labeling of the top 10 genes with the most significant p-values, as illustrated in Fig. 2. To address potential issues such as noise, redundancy, and batch effects, we applied the Combat function from the sva R package. This step harmonized the four datasets, aligning them to minimize discrepancies and improve overall data quality. By removing the batch effect, we verified that the corrected data set had a consistent expression pattern across batches and retained true biological differences. The outcomes of this harmonization process are depicted in Fig. 3, demonstrating the effectiveness of our approach in achieving more reliable and integrated results.

### Results of the RRA analysis

**Fig. 4:**
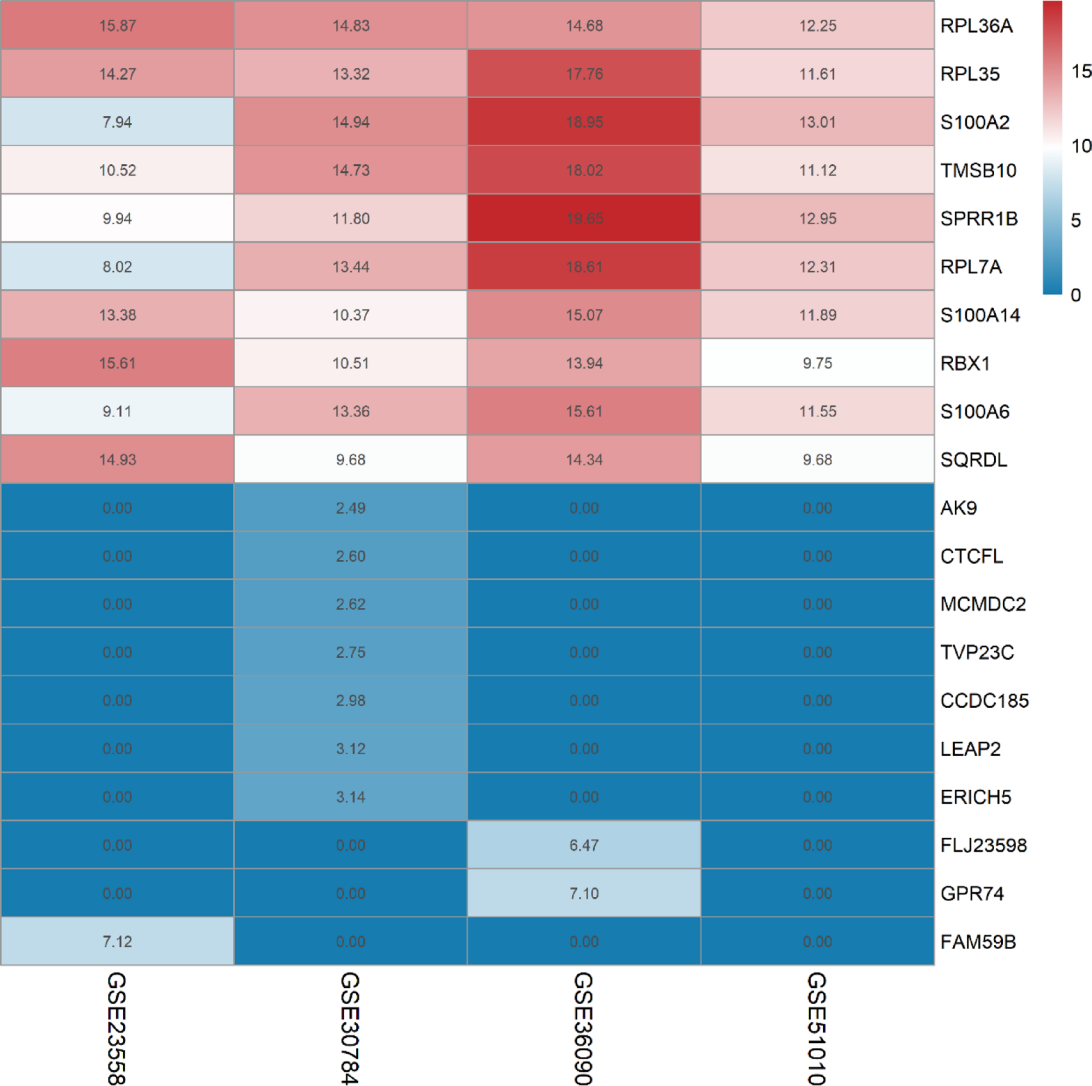
Heatmap of RRA analysis. We conducted a comprehensive analysis of the four datasets using the RRA method. This integrated approach allowed us to identify and rank the top 10 genes that exhibited significant up-regulation and down-regulation across each datasets. In the figure, the horizontal axis corresponds to the datasets, while the vertical axis displays the gene names. The data has been normalized by columns. Each box within the figure represents the log2 FC values of the respective genes.

In the RRA analysis, we applied a criterion of p-values less than 0.05 to identify differentially expressed genes, leading to the selection of 101 significant genes for further examination. To aid in the interpretation and subsequent research, we visualized the top 10 upregulated and top 10 downregulated genes separately. These visualizations, presented in Fig. 4, provide a clear and focused overview of the most prominent gene expression changes, facilitating a more detailed exploration of their roles and potential implications in the context of our study.

### Results of the WGCNA analysis

Weighted Gene Co-expression Network Analysis (WGCNA) is a robust method widely employed in disease research to uncover candidate biomarkers, elucidate disease mechanisms, and identify potential therapeutic targets. This technique organizes genes into co-expression modules, where it groups genes that exhibit similar expression patterns, thus highlighting clusters of highly correlated genes. This grouping is instrumental in discovering potential biomarkers associated with the disease. Moreover, by constructing and analyzing gene expression networks, WGCNA identifies key hub genes that play crucial roles in disease progression. These hub genes are often central to the network and can serve as critical targets for further investigation. Consequently, WGCNA provides valuable insights into the molecular underpinnings of diseases and offers promising strategies for the development of biomarkers and therapeutic interventions aimed at mitigating disease progression.

**Fig. 5:**
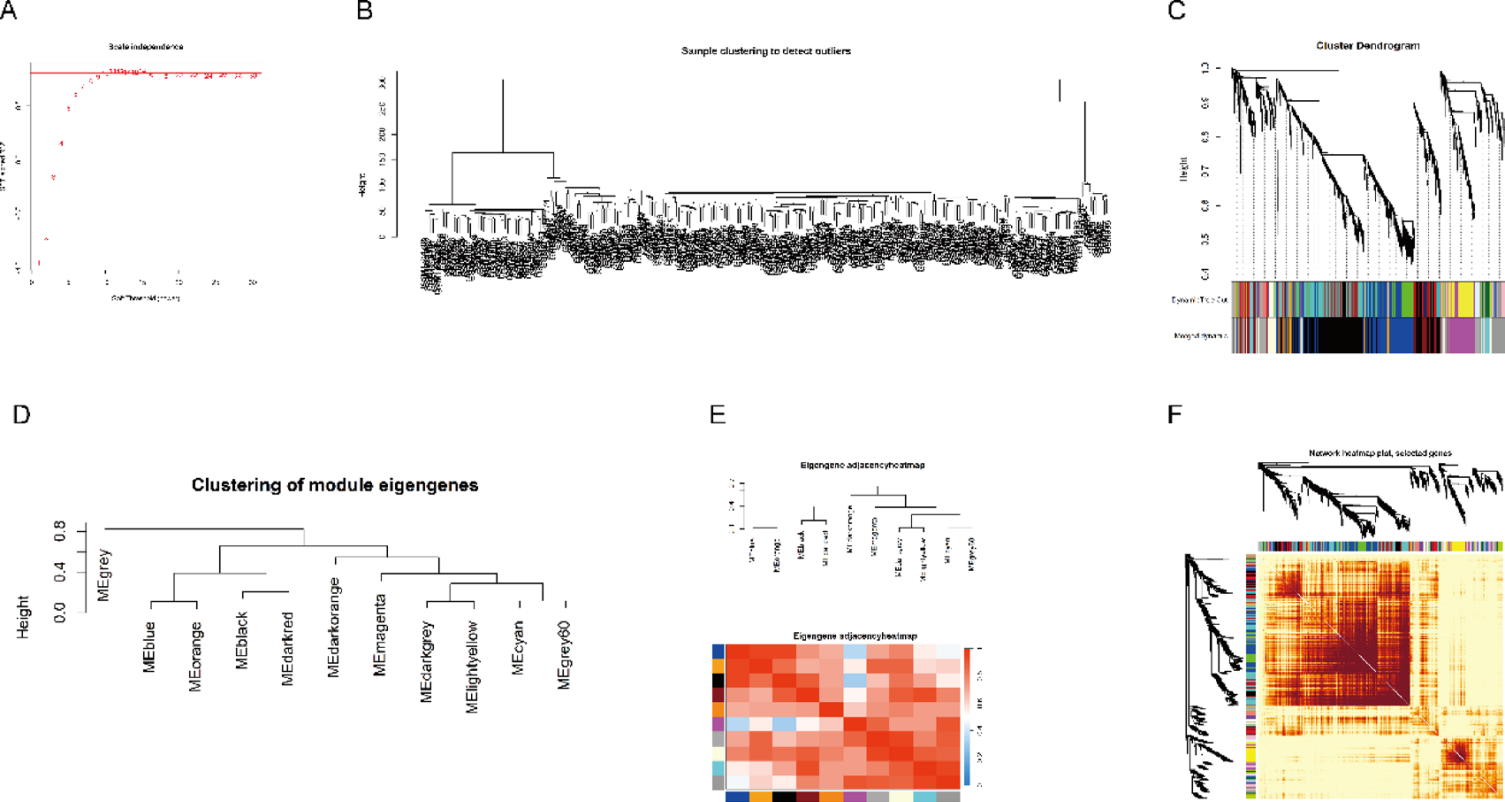
Results of the WGCNA Analysis. (**A**) Selecting suitable soft thresholding parameters is a crucial step in WGCNA analysis to ensure accurate network construction. (**B**-**E**) These panels illustrate the identification and characterization of gene modules within the WGCNA framework, showcasing how genes are grouped into distinct co-expression modules based on their expression profiles. (**F**) This figure depicts the partitioning of the entire gene expression matrix into these identified modules, demonstrating how the data is segmented into biologically relevant clusters for further analysis.

To explore the broad impact of OSCC on physiological functions, we applied WGCNA to gene expression matrices. Despite previous studies on gene function, many aspects remain unknown. Initially, we used the functions “goodSamplesGenes()” and “hclust()” to reduce outliers in the sample data. Subsequently, we employed the “pickSoftThreshold()” function to select an appropriate threshold matrix. Using clustering results and distance matrices, we applied the dynamic tree cut algorithm to identify modules and set minimum gene thresholds for each module. Modules were assigned colors using “labels2colors()” and module eigengenes were computed using “moduleEigengenes()” to assess inter-module correlations. Finally, genes with 90% similarity were merged into modules, and hub genes within each module were identified for further exploration, as depicted in Fig. 5.

### Results of the GO and KEGG analysis

**Fig. 6:**
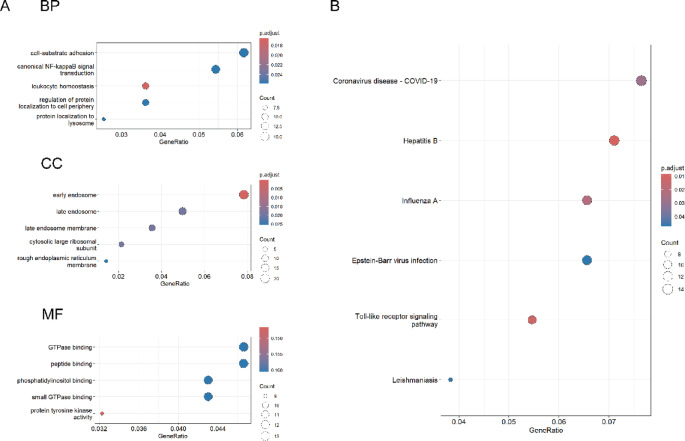
The GO and KEGG analysis of the integrated DEGs. We took the concatenated set of differentially expressed genes obtained from RRA and WGCNA and subjected these genes to GO analysis. (**A**)the top five pathways in terms of biological process (BP), cellular component (CC), and molecular function (MF) are shown. (**B**) show the top 10 pathways analyzed by KEGG.

Subsequently, we conducted Gene Ontology (GO) functional annotation and KEGG pathway enrichment analyses on the combined set of genes. This set includes 101 Differentially Expressed Genes (DGEs) identified through the Robust Rank Aggregation (RRA) analysis and 225 Differentially Expressed Genes (DEGs) detected by the Weighted Gene Co-expression Network Analysis (WGCNA). By integrating these two sets of differentially expressed genes, we aimed to gain a comprehensive understanding of their biological functions and the pathways they influence. The findings from these analyses are illustrated in Fig. 6, providing detailed insights into the functional roles and pathway associations of the identified genes.

## Discussion

In our study, we investigated potential biological targets for OSCC by analyzing four independent datasets. We employed integrated methodologies, including Robust Rank Aggregation (RRA) and Weighted Gene Co-expression Network Analysis (WGCNA), to identify critical genes and significant pathways related to the disease. Through RRA, we consolidated differential expression data to highlight key genes consistently associated with OSCC. Complementarily, WGCNA was utilized to map gene co-expression networks and uncover pivotal genes involved in disease progression. The combined insights from these approaches revealed new potential therapeutic targets and offered a deeper understanding of the molecular mechanisms underlying OSCC. These findings pave the way for future research and the development of targeted treatments aimed at improving patient outcomes.

SH3BP4 (SH3 Domain Binding Protein 4) is a protein-coding gene that interacts with SH3 domains. The SH3 domain is a small and commonly occurring domain crucial in signaling and intracellular communication. SH3BP4 interacts with SH3 domains of other proteins, participating in the regulation of various cellular processes^22–24^. SH3BP4 consists of two regions: the SH3 Binding Region and other functional domains. In organisms, SH3BP4 plays the following roles: (1) Signal regulation: It modulates cellular signaling pathways, potentially influencing pathway activity through interactions with other proteins^25^. (2) Cytoskeletal regulation: It plays a crucial role in the remodeling and dynamic regulation of the cytoskeleton, orchestrating changes in the structure and organization of the cytoskeletal framework. (3) Cell adhesion and migration: It may affect cell adhesion and migration capabilities, crucial processes in development and disease progression^26–28^. Studies suggest SH3BP4 may be implicated in tumorigenesis and progression. For instance, its dysregulation could impact cell proliferation and migration, thereby promoting tumorigenesis. In a separate research investigation, the discovery of Single Nucleotide Polymorphisms (SNPs) within the SH3BP4 gene revealed an intriguing overlap with predicted microRNA binding regions. This phenomenon suggests a plausible scenario where these SNPs could interfere with multiple miRNA-mRNA interactions, influencing the prognosis of patients with laryngeal cancer. This intricate interplay between genetic variations within SH3BP4 and miRNA-mediated regulatory mechanisms may have significant implications for understanding the molecular basis of laryngeal cancer progression and overall patient survival outcomes. Further exploration of these interactions could potentially unveil novel therapeutic targets and personalized treatment strategies in the context of laryngeal cancer management^29^. Consequently, SH3BP4 emerges as a promising candidate for both early detection and therapeutic intervention in OSCC. Its potential as a biomarker for early diagnosis is underscored by its involvement in key molecular pathways related to the disease, offering a valuable tool for identifying cancer at an earlier, more treatable stage. Additionally, SH3BP4’s role in disease progression and its interaction with critical regulatory networks position it as a viable therapeutic target. Targeting SH3BP4 could lead to the development of novel treatments aimed at disrupting cancer-specific pathways, potentially improving treatment efficacy and patient outcomes in OSCC. Studies have shown that the loss of SH3BP4 increases the number of intestinal stem cells and Paneth cells in mice, and accelerates the development of adenomas in Apc^min^ mice^26^. Interestingly, other studies have found that SH3BP4 is a protein-binding partner of the protein GIPC1, and GIPC1 knockdown reduces endogenous MACC1 expression and reduces MACC1-induced cell migration and invasion^30^. In a cohort study, the authors identified the tumor suppressor SH3BP4 as a biomarker for melanoma using whole-set genomic association analysis^27^. In another sequencing study, the authors found that SH3BP4 was down-regulated in glioblastoma cultured under neuroglobular formation conditions^31^. Further research into SH3BP4’s functions and interactions will be crucial for validating its utility in clinical settings and optimizing therapeutic strategies.

RRAGC (Ras Related GTP Binding C) encodes a small GTP-binding protein that is a member of the Rag GTPase family. This gene is integral to cellular processes involving nutrient sensing and signal transduction. The RRAGC protein comprises multiple functional domains, including a GTP-binding domain and specific effector-binding regions. Its structure resembles other Rag GTPases, featuring an N-terminal GTPase domain and a C-terminal effector-binding domain^32,33^. In conjunction with RRAGA, RRAGC is instrumental in regulating the localization and activity of the mTORC1 complex. This regulation is crucial for overseeing essential physiological processes including cellular metabolism, proliferation, and growth. RRAGC significantly influences how cells respond to amino acid availability by modulating the signaling pathways of mTORC1. Through its role in these signaling cascades, RRAGC helps ensure that cellular functions are appropriately adjusted according to nutrient levels, thereby maintaining cellular balance and promoting optimal growth and metabolic efficiency. This dynamic regulation underscores RRAGC’s importance in adapting to fluctuating nutrient conditions and its impact on broader physiological processes. RRAGC forms a complex with RRAGA as part of the Rag GTPase complex, regulating mTORC1 activation state through GTP hydrolysis^33–38^. Studies have shown that RRAGC-activating mutations are found in approximately 10% of follicular lymphomas, with the mutated RRAGC protein showing increased binding to RPTOR (raptor) and significantly reduced interaction with the product of the tumor suppressor gene FLCN (follicle protein). It is suggested that RRAGC is associated with the occurrence of cancer^39–41^. Additional studies have found that MTOR (mechanistic target of rapamycin kinase) complex 1 (MTORC1) coordinates different environmental signals to promote cell growth and is frequently activated in cancer. The translocation of MTORC1 from cytosol to lysosome surface by RRAGC GTase is a key step in MTORC1 activation, and inhibition of RRAGC expression can also inhibit the growth of cancer cells^42^. Hence, RRAGC regulates cancer occurrence and may be a key gene in the modulation of OSCC.

SQRDL (Sulfide quinone reductase-like) is a protein gene involved in cellular metabolic regulation and redox reactions. It typically consists of functional domains, potentially including domains interacting with sulfides or quinoline compounds, as well as domains facilitating electron transfer. The protein encoded by this gene is likely localized within the mitochondria, where it plays a critical role in mitigating sulfide toxicity. It accomplishes this by catalyzing the biochemical conversion of sulfides into thiosulfates. This enzymatic process helps to neutralize potentially harmful sulfide compounds, thereby protecting mitochondrial function and cellular health. By efficiently managing sulfide levels, this protein contributes to the maintenance of cellular homeostasis and prevents the accumulation of toxic substances that could impair mitochondrial performance and overall cellular well-being^43–47^. Alternative splicing produces several transcript variants that all encode the same protein. SQRDL is essential for proper skeletal development and has been closely linked to osteoporosis, particularly in postmenopausal women. This gene’s alternative splicing may lead to different isoforms of the protein, potentially affecting its function and contribution to bone health. SQRDL’s involvement in skeletal development underscores its importance in bone formation and maintenance, while its association with osteoporosis highlights its potential role in the pathogenesis of this condition^48,49^. Studies have shown that SQRDL is down-regulated in rectal cancer, and unfortunately, the authors did not further explore its biological function^50^. In another study, the authors integrated brain proteomics and transcriptomic data to find a causal relationship between SQRDL and sleep apnea^51^. Despite its known role in skeletal development and association with osteoporosis, there is currently a significant gap in research regarding the connection between SQRDL and OSCC. To fully understand whether and how SQRDL might influence the development or progression of OSCC, further biological experiments are required. These investigations should focus on elucidating the potential roles of SQRDL in the context of oral cancer, examining its expression levels, functional impacts, and possible mechanisms of action. Such studies could provide valuable insights into whether SQRDL is involved in the pathogenesis of OSCC and could uncover novel aspects of its biological functions, potentially leading to new diagnostic or therapeutic approaches for managing OSCC. Understanding this connection could potentially shed light on novel therapeutic strategies targeting SQRDL in cancer treatment contexts.

There are many risk factors for OSCC, and HPV infection is one of them. HPV, a compact DNA virus, exhibits a pronounced affinity for squamous epithelial cells. To date, researchers have identified 202 distinct HPV variants. Mucosal-infecting HPV strains are categorized into high-risk and low-risk groups, depending on their capacity to induce disease. Low-risk strains, including HPV6 and HPV11, are typically associated with non-cancerous wart-like growths. In contrast, high-risk strains, such as HPV16 and HPV18, are strongly linked to the development of precancerous changes in squamous epithelial tissue and have the potential to progress to invasive cancers^52,53^. In summary, by combining data from four datasets through the RRA and WGCNA algorithms, we successfully identified differentially expressed genes (DEGs) linked to the development of OSCC. This integrated approach provides valuable insights that could support the diagnosis and treatment of OSCC. Additionally, our GO functional annotation and KEGG pathway enrichment analyses highlighted key signaling pathways enriched among these DEGs. However, the precise mechanisms by which these DEGs contribute to the carcinogenic process in OSCC remain uncertain. Further research is needed to clarify the roles of these DEGs and validate their potential as biomarkers or therapeutic targets in OSCC.

We identified several key genes that are closely related to OSCC progression. These genes play an important role in the development and development of OSCC. From previous evidence, these key genes are involved in rectal cancer, follicular lymphoma, melanoma, etc., suggesting that they may also be involved in the development of OSCC. SH3BP4 is also involved in cell migration and invasion, suggesting that it may be involved in remodeling the tumor microenvironment as well as immune escape mechanisms. From a clinical perspective, the expression levels of these genes could serve as novel biomarkers for early diagnosis, prognostic assessment, and the formulation of individualized therapies. Through comparative analysis of gene expression profiles of patients with different clinical stages and subtypes, it was found that high expression of certain genes was associated with poor prognosis, and could predict clinical outcomes such as survival and recurrence risk of patients. Therefore, monitoring the expression level of these genes can not only provide important information for the early detection of tumors, but also serve as a basis for stratified treatment. Previous translational medicine research has shown that some key genes can be used as potential targets for targeted therapy. Gene suppression or activation may improve patient outcomes by interfering with cell proliferation or inhibiting the tumor’s ability to metastasize. In addition, gene detection technology can be used for patient screening to help select the appropriate treatment plan and improve the accuracy and effectiveness of treatment. Future studies can further analyze the function of these genes to reveal their role in tumor initiation, progression, and metastasis, leading to the development of novel diagnostic tools and therapeutic strategies. In addition, based on the expression patterns of these genes, researchers can evaluate their value in clinical applications, such as as biomarkers for early screening, prognostic assessment, and treatment response prediction. In the future, the clinical value of these genes will be verified through multi-center clinical studies, combined with multi-dimensional data such as genomics and proteomics, which is expected to provide more comprehensive support for OSCC’s precision medicine.

### Limitation

In this study, we systematically searched the GEO database for all relevant datasets compared with OSCC patients and healthy individuals. However, after screening, we found only four data sets that met the research criteria. This leads us to face relatively small data volume limitations in our analysis, which can affect the broad applicability and statistical power of our findings. In addition, when we selected the data set to be included in this study, we selected the microarray data of human oral squamous cell carcinoma and healthy samples with strong homogeneity. Due to the different years of each sequencing, there may be certain errors in the calculation results. Due to this study mainly relies on bioinformatics analysis, and we currently lack the appropriate experimental conditions, the key genes selected have not been experimentally validated. Nonetheless, all of the key genes screened were supported by multiple public data sets and existing literature, with strong biological plausivity and clinical relevance. We recognize the importance of experimental validation, so in future studies, biologists can build on the findings of this study to further validate the function and clinical application potential of these genes in OSCC.

## Data Availability

The raw data for all four datasets involved in the manuscript are available in the GEO database, https://www.ncbi.nlm.nih.gov/.
